# Life-Threatening Retroperitoneal Hematoma in a Patient with COVID-19

**DOI:** 10.1155/2021/8774010

**Published:** 2021-11-05

**Authors:** Monarch Shah, John Paul Colombo, Sanya Chandna, Haris Rana

**Affiliations:** ^1^Department of Internal Medicine, Saint Peter's University Hospital, 1050 George Street, Apartment 4i, New Brunswick, NJ 08901, USA; ^2^Department of Pulmonary and Critical Care Medicine, Saint Peter's University Hospital, 1050 George Street, Apartment 4i, New Brunswick, NJ 08901, USA

## Abstract

COVID-19 is a respiratory illness that affects the human body in many different ways. The disease carries both thrombotic and hemorrhagic complications, especially in those patients who are anticoagulated to prevent the thromboembolic manifestations. In this report, we discuss a case of retroperitoneal hemorrhage in a patient treated with therapeutic anticoagulation which ultimately led to the patient's death. The literature highlights the importance of anticoagulation because it reduces mortality in patients hospitalized with COVID-19. Although, more recent studies suggest that patients treated with therapeutic anticoagulation are at a higher risk of hemorrhage and increased mortality. Therefore, our case stresses the importance of active monitoring of these patients to detect any suspected case of hemorrhage early to reduce mortality. Overall, more research should be conducted to determine the optimal dosing of anticoagulation that balances safety and efficacy.

## 1. Introduction 

COVID-19 is associated with thrombotic complications and increased mortality. It may be necessary to give anticoagulation to every hospitalized patient with COVID-19; however, we know that the bleeding risk is increased in those with COVID-19. Physicians must be very cautious when it comes to using prophylactic anticoagulation and carefully assess the patients' status and laboratory values when it comes to deciding to increase the dosage to therapeutic anticoagulation. This report amongst others highlights the importance of continued active surveillance of the patients' status and symptoms. In addition, this report stresses the need for clinical trials to determine the optimal dosage of anticoagulation that is not only effective but also safe in the treatment of COVID-19 thrombosis. Further studies will also be needed to tease out risk factors that would categorize a patient as high, intermediate, or low risk for hemorrhage when using a specific dosage of a particular anticoagulant. Until then, we hope to add to the knowledge that enables us to think about hemorrhagic complications when using anticoagulation in COVID-19 patients.

## 2. Case Presentation

**Figure 1:**
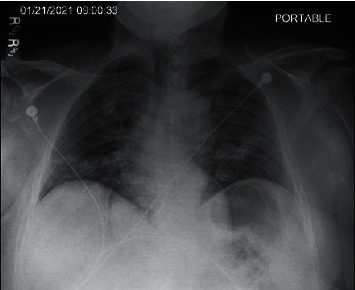
Chest X-ray: patchy bilateral infiltrates.

A 67-year-old man was admitted with a complaint intermittent shortness of breath for 2 weeks and a dry nonproductive cough for 1 week. He also had associated fever, chills, and fatigue. He had tested COVID-19 +ve at an urgent care clinic 3 days before admission and was prescribed an Albuterol inhaler and a 3-day course of oral Azithromycin. His past medical history was significant for hypertension, hyperlipidemia, type 2 diabetes mellitus, coronary artery disease with a history of stent placement in 2005, currently taking only aspirin for it, nephrolithiasis, and gout. There was no history of smoking, alcohol, or recreational drug use. He was allergic to contrast dye and had an unknown allergic reaction after the previous cardiac catheterization he had. On arrival, his temperature was 98.9 F, blood pressure was 129/96 mm of hg, and heart rate was regular at 105/minute, respiratory rate was 24/minute and he was saturating at 92–95% on 2 liters of oxygen via a nasal cannula. Pertinent positive physical examination findings included crackles in bilateral lower lung zones. Cardiac examination was normal, and he had no lower extremity edema or calf tenderness. Relevant laboratory findings were significant for an elevated white blood cell count of 17.2 × 10^3^/mm^3^ (4–11 × 10^3^/mm^3^), 90% of which were neutrophils, hemoglobin of 14.6 g/dl, platelet count of 236 × 10^3^/mm^3^, absolute lymphocyte count 0.34 × 10^3^/mm^3^ (1–3.5 × 10^3^/mm^3^), blood urea nitrogen 32 mg/dl (9–28 mg/dl), creatinine 1.96 mg/dl (0.66–1.25 mg/dl), glomerular filtration rate 34 ml/min/1.73 m^2^, creatine clearance 53 ml/min, D-dimer 437 ng/ml (0–211 ng/ml), lactate dehydrogenase 343 U/L (140–271 U/L), and C-reactive protein of 145.7 mg/L (0–5 mg/L), and the SARS-CoV-2 RNA polymerase chain reaction test was positive. The patient's 4 C mortality score was 13 points putting him at high risk of inpatient mortality. Chest X-ray (Figure 1) showed patchy bilateral infiltrates consistent with bilateral pneumonia.

**Figure 2:**
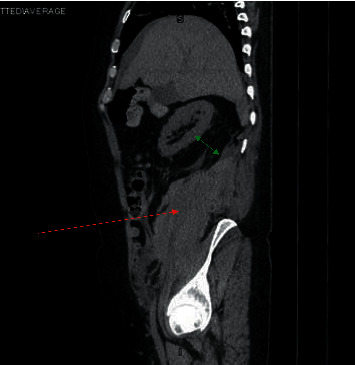
CT abdomen pelvis sagittal view: large right-sided retroperitoneal hematoma extending along the psoas musculature (red arrow) and the lateral abdominal wall with areas of high attenuation. The right kidney is displaced anteriorly by the retroperitoneal hematoma (green arrow).

**Figure 3:**
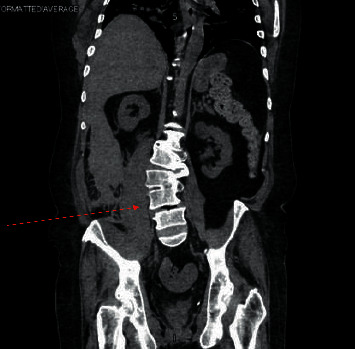
CT abdomen pelvis coronal view: large right-sided retroperitoneal hematoma extending along the psoas musculature and the lateral abdominal wall.

Treatment was started with oral dexamethasone 6 mg daily to be given for 10 days, and he was given anticoagulation with enoxaparin 40 mg subcutaneously daily. Remdesivir was not indicated at that point as the patient was more than 2 weeks from symptom onset. Proning was encouraged, and the patient's oxygenation was being monitored closely with a goal for oxygen saturation being >90%. On day 2 of hospitalization, the patient continued to be dyspneic and his oxygen requirements were varying from 2–4 liters/minute with a drop to less than 90% with ambulation on 3 liters/minute oxygen via a nasal cannula. On day 3 of hospitalization, the oxygen requirements increased to 5 liters/minute via nasal cannula oxygenation at rest and on ambulation, and clinically, the patient's symptoms did not improve. Due to worsening hypoxia and unimproved clinical status, his anticoagulation was increased to a therapeutic dose of 100 mg every 12 hours. The next day, the patient continued to have a dry cough, shortness of breath, and intermittent pleuritic chest pain but was also now complaining of pain in the lower back, mainly on the right side and radiating to his groin. Owing to his history of nephrolithiasis, there was a suspicion for the same, but due to the recent increase in the dose of anticoagulation, the possibility of intrabdominal bleeding could not be ruled out. Due to the history of contrast allergy, a computerized tomography (CT) of the abdomen and pelvis (Figures 2 and 3) was performed that showed a large right-sided retroperitoneal hematoma extending along with the psoas musculature and the lateral abdominal wall with areas of high attenuation and the right kidney was displaced anteriorly because of the same, Table 1.

Upon reevaluation of the patient, his blood pressure was 136/80 mm of Hg, heart rate was regular at 90/minute, and there were no changes in oxygen requirement. He continued to complain of low back pain. Hemoglobin drawn at that time was 13.2 which was unchanged from the day before. Antifactor Xa levels were not drawn up until that point. At that point, the plan was to closely monitor the patient with serial abdominal examination and hemoglobin checks and no further anticoagulation. Within 2 hours of the CT scan, the patient was found to be unresponsive and pulseless following which a code blue was called and despite attempting resuscitation for more than 30 minutes, the patient passed away.

## 3. Discussion

COVID-19 is a respiratory illness causing symptoms from mild to life threatening in different individuals. However, the SARS-CoV-2 virus's effects on the human body are not limited to the respiratory system. In a Nature review article, the extrapulmonary manifestations of COVID-19 are highlighted along with the proposed pathophysiologic mechanisms and management considerations for each system [1]. Current evidence suggests that 16–49% of admitted ICU patients with COVID-19 suffer from venous thromboembolism [2]. The laboratory studies in COVID-19-associated coagulopathy tend to show elevated levels of D-dimer and fibrinogen, minor elevations in prothrombin time, activated partial thromboplastin time, and decreased platelet count in the initial stage of disease [1, 2]. These changes are said to be due to an interplay of cytokine-induced inflammatory reaction and direct viral entry into endothelial cells causing endothelial inflammation, as well as upregulation of ACE-2 receptors on endothelial cells perpetuating further damage [1–3]. In addition, the viral-induced elevation of von Willebrand factor, Toll-like receptor activation, and tissue factor activation lead to extrinsic coagulation pathway perpetuation activation and subsequent formation of fibrin clots [2–4]. This thromboinflammatory state contributes to micro- and macrovascular thromboses such as deep venous thrombosis, pulmonary emboli, and pulmonary intravascular coagulation which further contribute to hypoxemic respiratory failure in the setting of COVID-19 or ARDS [2].

To prevent and treat the thrombotic complications of COVID-19, physicians have been using prophylactic and therapeutic dosing of unfractionated and low-molecular-weight heparin (LMWH) in attempts to evade coagulopathies [2]. Current guidelines for managing coagulopathy in COVID-19 patients have been set by the American Society of Hematology (ASH), the International Society of Thrombosis and Haemostasis (ISTH), and the American College of Cardiology [2]. The recommendations are to give all hospitalized COVID-19 patients a dose of LMWH in the absence of active bleeding or except when the platelet count is <25 × 10^9^/L or fibrinogen levels is <0.5 g/L. Abnormalities in PT or aPTT are not a contraindication to thromboprophylaxis, and when prophylaxis is contraindicated, mechanical thromboprophylaxis should always be used [2]. These guidelines are based upon evidence that elevated D‐dimer levels are associated with increased mortality in COVID‐19 patients. Also, multiorgan failure is more likely in septic patients that develop coagulopathy. Therefore, thromboprophylaxis may reduce mortality [2]. In fact, there is evidence that anticoagulated COVID-19 patients have a decreased mortality and decreased rates of intubation than those not anticoagulated [5].

However, there has been much debate about whether to use anticoagulants in certain circumstances and what dosage should be used [2]. Although anticoagulation may reduce complications of COVID-19 and reduce its mortality, there is also evidence to suggest that anticoagulation may lead to life-threatening hemorrhage. In a retrospective study, there were significantly higher rates of bleeding and higher rates of inpatient mortality in patients on therapeutic anticoagulation [6]. The most common site of hemorrhage tended to be outside of the central nervous system and gastrointestinal tract; however, every patient with an intracranial bleed died [6]. There are many other reports of hemorrhagic complications in anticoagulated COVID-19 patients such as cases of intracranial, gastrointestinal, intramuscular, and retroperitoneal bleeds [7–12]. There is also evidence of hemorrhagic events in patients who did not receive anticoagulation [8, 13]. Our case is very similar to other case reports in which patients who were anticoagulated either prophylactically or therapeutically developed retroperitoneal hemorrhage [7, 9–12]. It could be hypothesized that microvascular vulnerability derived from atherosclerosis, microtrauma due to COVID-19, and mechanical disruption, including cough, could lead to the retroperitoneal hemorrhage we are seeing in these patients [9]. In some patients, retroperitoneal hemorrhage was discovered early enough through CT angiography and was able to be treated with arterial embolization whereas other patients, such as ours, were not treated in time, leading to their death. This highlights the importance of active surveillance of COVID-19 patients, especially those being anticoagulated. Physicians must keep an eye out for hypovolemic signs, anemia, and symptoms such as vague abdominal or flank pain to detect hemorrhage early. If hemorrhage is suspected, CT angiography should be ordered immediately to rule out life-threatening hemorrhage.

## Figures and Tables

**Table 1:** Pertinent laboratory investigations during hospital course.

Laboratory values (reference ranges)	On admission	Day 3 (worsening oxygen requirements)	At the time of development of hematoma
Hemoglobin (13.0–17.0 g/dL)	14.6	13.8	13.2
Platelet count (150–400 × 10^3^ mm^3^)	236	226	397
Prothrombin time (10.4–13.7 sec)	11.9	12.8	12.2
Activated partial thromboplastin time (27.2–35.7 sec)	26.1	33.2	31
D-dimer (0.00–211.00 ng/mL)	437	554	521

## Data Availability

The literature review data used to support the findings of this study are included within the article.
